# Evaluation of Self-Propelled Rotary Tool in the Machining of Hardened Steel Using Finite Element Models

**DOI:** 10.3390/ma13225092

**Published:** 2020-11-11

**Authors:** Usama Umer, Hossam Kishawy, Mustufa Haider Abidi, Syed Hammad Mian, Khaja Moiduddin

**Affiliations:** 1 Advanced Manufacturing Institute, King Saud University, Riyadh 11421, Saudi Arabia; mabidi@ksu.edu.sa (M.H.A.); smien@ksu.edu.sa (S.H.M.); khussain1@ksu.edu.sa (K.M.); 2 Machining Research Laboratory, University of Ontario Institute of Technology, Oshawa, ON L1G 0C5, Canada; Hossam.Kishawy@uoit.ca

**Keywords:** finite element modeling, hard turning, self-propelled rotary tool, tool wear, chip flow, cutting force

## Abstract

This paper presents a model for assessing the performance of self-propelled rotary tool during the processing of hardened steel. A finite element (FE) model has been proposed in this analysis to study the hard turning of AISI 51200 hardened steel using a self-propelled rotary cutting tool. The model is developed by utilizing the explicit coupled temperature displacement analysis in the presence of realistic boundary conditions. This model does not take into account any assumptions regarding the heat partitioning and the tool-workpiece contact area. The model can predict the cutting forces, chip flow, induced stresses, and the generated temperature on the cutting tool and the workpiece. The nodal temperatures and heat flux data from the chip formation analysis are used to achieve steady-state temperatures on the cutting tool in the heat transfer analysis. The model outcomes are compared with reported experimental data and a good agreement has been found.

## 1. Introduction

The innovations in material science contribute to the emergence of new materials. These modern materials exhibit peculiar mechanical and thermal properties. Such materials, due to their remarkable properties have numerous applications in high-performance industries such as the biomedical sector, sensors, aerospace, automobile, etc. Nevertheless, on the one hand, the excellent properties of these materials are advantageous, on the contrary, they pose obstacles and difficulties in the machining of these materials. The serious challenge that arises when such materials are being machined is intense heat emission which in turn influences the wear and machining efficiency of the tool [1,2,3].

Several methods have been practiced to address the problem of elevated heat generation, such as using liquid coolant in the machining zone [4,5,6]. The temperature at the cutting zone is kept within the appropriate limits through the assistance of coolants. However, there are some concerns related to coolants, such as not being eco-friendly, maintaining their proper flow rate during machining, as well as selecting the best coolant for a specific material, etc. Amongst the various current techniques, the use of rotary cutting tools is the most versatile and reliable approach to resolve the challenge of excessive heat generation [7]. When difficult to cut materials are machined under dry conditions, rotary tools offer an effective solution to the problem of extreme heat dissipation while preserving the satisfactory tool performance [8]. For instance, in [9,10], Kishawy et al. stated that rotary tools substantially enhanced the tool life, minimized the cutting temperature, and increased the Material Removal Rate (MRR). Ezugwu [11] also acknowledged what has been documented in [9,10], as well as asserted that rotary tools produced smoother surface particularly in the case of materials that were difficult to cut. Therefore, the rotary tools have considerably gained the attention of the machining community because of their superior performance for hard-to-machine materials. 

The rotary cutting tool can be described as a tool that portrays the shape of a circular disk and that rotates around its center. Compared to the traditional turning tool in which a single cutting point is utilized, the rotary tool rotates around its center during machining and the cutting process takes place throughout the cutting edge perimeter (i.e., circular circumference) owing to this rotation. There are primarily two kinds of rotary tools that have been described in the literature, namely Actively Driven Rotary Tool (ADRT) and Self-Propelled Rotary Tool (SPRT) [12]. The tool based on the principle of ADRT rotates using an external motor, so the tool rotation speed and direction (in the direction of the clockwise or anti-clockwise direction) can be regulated. Furthermore, the angle of inclination which is the most dominant factor influencing the speed of the tool can be adjusted to any value as the motion of the tool is controlled through an external power supply. In contrast, the SPRT rotates around its axis (i.e., chip friction force) due to the contact between the tool and the workpiece [13]. Though, ADRT can provide more control but necessitates additional power source which renders it complex and expensive. The SPRT, on the other hand, is an economical and feasible option for the effective cutting of materials that are hard to machine [14]. 

Various researches can be listed out in the literature that have carried out experimental studies to examine the performance of SPRT in machining difficult to cut materials. For example, Chen et al. [15] published experimental results for machining the SiC_w_/Al composite by employing carbide rotary cutting methods, particularly SPRT. Their findings showed that the tool life of rotary tools was considerably greater than that of the traditional tools. Similarly, the consequences of SPRT were investigated during the machining of aerospace material (Titanium IMI 318 alloy) [16]. The analysis revealed that the cemented carbide SPRT increased the tool life sixty times in contrast to round and rhomboid inserts utilized in single-point turning. However, when the Inconel 718 alloy was machined using a chemical vapor deposited coated carbide SPRT, the enhancements in tool wear were not as meaningful as opposed to the cemented carbide SPRT. In yet another research, Kishawy and Wilcox [17] conducted a SPRT performance analysis while hardened steel was being machined. They examined the chip morphology and the wear of the tool. The SPRT carbide tool displayed tremendous wear resistance and there was no crater wear, as well as the flank wear was uniformly distributed. Kishawy et al. [9] also introduced an experimental analysis to evaluate the SPRT and surface quality. The workpiece material used was an aerospace-grade titanium alloy and nickel-based alloys. The elevated performance of SPRT was noted in this study when contrasted with a single point tool having the same angle and tool profile (circular). 

El-Mounayri et al. [18] administered an exploratory study to compare the cutting force, tool wear, and quality of the machined surface between SPRT and traditional tools. The turning of the difficult to process 55RC workpiece material was executed through carbide coated titanium nitride inserts. The findings in this study demonstrated the expanded tool life and higher rotary tool efficiency. Kossakowska and Jemielniak [19] also researched the usefulness of SPRT for machining of the 15H11MF high alloy steel. Some of the impediments of SPRT (e.g., twirling the chips through the rotating rake face of the tool) and advantages (such as better life of the tool) were published. Undoubtedly, these studies have illustrated the effectiveness and viability of SPRT for machining difficult-to-cut materials. Perhaps it can be acknowledged based on the literature that SPRT provides many advantages when processing materials that are hard to machine. 

There are several factors, namely cutting speed, depth of cut, feed rate, tool wear, tool geometry, etc., that can affect the quality of SPRT machining. Consequently, numerous studies examining the different variables can also be highlighted in the literature. For example, Armarego and Katta [20] established a predictive model for forces and power during the machining of S1214 steel by deploying TiN-coated and uncoated carbide SPRT. Their research demonstrated that the coating slightly decreased the vertical force and thus, the power with greater reductions in the feed. In another study, a carbide SPRT model for cutting AISI4340 steel with 54–56 HRC hardness was presented [10]. The model was formulated for flank wear while utilizing SPRT. The results revealed that the developed model predicted the flank wear quite effectively. Likewise, Hao et al. [21] introduced a model for SPRT to measure the cutting forces by using a combination of Artificial Neural Network (ANN) and the Genetic Algorithm (GA). The specimen employed in the study was a low carbon steel plate, while the feed rate, cutting velocity, cutting depth, and tool inclination were the input variables. The established algorithm responded well when results were confirmed through experiments. The mathematical model introduced by Kishawy et al. [22] effectively measured the chip-flow for the SPRT. The acquired model demonstrated a strong agreement with the outcomes of the experiment. Similarly, Li and Kishawy [23] also built up a model for calculating the force when using SPRT in an oblique carbon steel cutting of SAE1045. The inserts employed were fabricated utilizing carbide (uncoated), with a diameter of 27 mm, a rake angle of 0°, and a 0° flake angle. The developed force model was assessed through experimental tests, and the predicted values were similar to the experimental ones. 

Hao et al. [24] described an autoregressive model to analyze the occurrence of vibration and chatter while using SPRT during machining. The results of this research disclosed that excessive vibrations were produced when the principal cutting power was based on a high frequency. Additionally, more chatter was noticed when a negative inclination was fixed at −40° or the positive angle of inclination was 70°. Olgun and Budak [25] measured the cutting forces, tool life, surface quality, and dimensional accuracy when using rotary and stationary tools in machining 1050 steel waspaloy and Ti6Al4V. The validation experiments confirmed the superior performance of the rotary tool. Many researchers have also analyzed the effectiveness of SPRT in turning the EN24 steel operation [26]. The machining variables were streamlined using the Design of Experiment (DOE) approach and the Non-Dominated Sorting Genetic Algorithm-II (NSGA-II). For example, Gurgen et al. [27] conducted a multi-response optimization, while using SPRT for turning the EN24 steel operation. NSGA-II was used to amend the surface roughness and MRR in combination with the Technique for Order Preference through Similarity to Ideal Solution (TOPSIS). Kishawy [28] also published an exhaustive analysis of SPRT and emphasized the significance of choosing suitable machining and tool geometry parameters for the superlative performance of SPRT.

Although, multiple experimental studies and mathematical models related to the use of SPRT, its variables, advantages, and disadvantages have been discussed in earlier works. However, there is a very limited number of studies in the literature that have utilized Finite Element (FE) for SPRT analysis. For example, a research work modeled the distribution of the tool temperature in SPRT when hardened steel was machined [8]. The FE analysis was implemented, and the moving heat source principle of conduction was utilized. This model was experimentally tested by using an infrared camera to estimate the temperature profile. Takahashi et al. [29] also addressed the effect of speed on tool life and tool temperature through FE to streamline the cutting conditions of the rotary tools. They examined the impact of speed ratio on the cutting force, temperature dispersion of chip, chip flow direction, chip thickness, etc. Likewise, FE was applied by Lotfi et al. [30] to demonstrate that the tool-chip contact time can considerably inhibit wear propagation on tool faces during the rotary motion of the cutting tool. They recorded minimal heat transmission from the chip to tool due to disengagement by employing the heat analysis. Furthermore, Lotfi et al. [31] implemented FE for conventional, rotary, and ultrasonic-assisted rotary tools to visualize tool wear and heat spread. They reported that the rotary cutting movement effectively impaired heat concentration in the contact area, as opposed to normal tool machining. 

The apparent disinclination to implement FE can be attributed to the difficulties in SPRT modeling and complexities inherent in its machining process. Certainly, it is imperative to introduce FE models that can figure out machining conditions depending on the different machine settings due to the significant expense of actual machining with rotary tools. The evolution of an accurate and precise FE model is essential to understand the machining mechanism of the rotary tools and to further improve their efficiency for difficult to machine materials. 

In this study, a three-dimensional (3D) FE model has been accomplished to simulate the formation of chips, while using coated carbide SPRT for AISI 51200 hardened steel machining. A coupled temperature displacement analysis is conducted using ABAQUS/Explicit. This model can predict different performance measures, such as cutting forces, chip morphology, stresses, and temperature in the workpiece and the cutting tool. Since the coupled temperature displacement analysis is computationally costly, this model cannot be used to predict steady-state temperatures on the cutting tool. Therefore, the heat transfer analysis is carried out using ABAQUS/STANDARD to model the steady-state temperatures on the cutting tool and the heat flux. The boundary conditions are incorporated using the outputs from the chip formation analysis. Finally, the model developed is validated concerning cutting forces using the outcomes reported in Dessoly et al. [8].

## 2. Finite Element Model 

**Figure 1:**
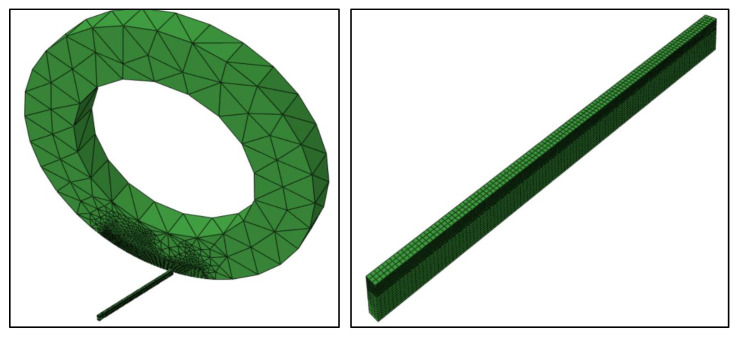
Workpiece and tool interaction (**left**) and workpiece mesh (**right**).

Figure 1 indicates the undeformed mesh for the tool and workpiece. A relatively fine mesh has been used on the tool edge and on the upper side of the workpiece to manage the higher gradients in the output variable across the region of interest. On the other hand, the coarse mesh is used for the remainder of the component regions to minimize the computation time.

The continuum elements with a temperature degree of freedom are preferred for the tool and workpiece. A total of 14112 elements are created using selected mesh schemes on the tool and workpiece. Table 1 lists the material properties of the workpiece and the cutting tool.

The identical cutting tool geometry as well as the process variables (as illustrated in Table 2) are adopted both in the FE model and the experiments. The boundary conditions are imposed such that the cutting tool is locked without the translation movement and the workpiece travels towards the cutting tool with the specified speed, as shown in Figure 1. The tool is permitted to move freely only around its central axis in case of tool rotation. In the fixed tool scenario, all rotational axis movements are not allowed. The ends of the tool face and workpiece are positioned as per the angles of inclination mentioned in the study.

The workpiece is simulated as an elastic-plastic material which includes the hardening strain rate and thermal softening effects. For this reason, the Johnson-Cook (J-C) model is chosen which is frequently found in applications of high strain rate deformation such as the metal cutting. The flow stress of J-C can be defined using Equation (1):

(1) $$\sigma =\left(A+B\varepsilon^{n}\right)\left(1+C\;\ln\left(\frac{\dot{\varepsilon }}{{\dot{\varepsilon }}_{0}}\right)\right)\left(1-{\left(\frac{T-T_{tr}}{T_{m}-T_{tr}}\right)}^{m}\right)$$

where *A*, *B*, *C*, *n*, and *m* are the material constants and they can be computed experimentally. ε is the equivalent plastic strain, $\dot{\varepsilon }$ is the equivalent plastic strain rate, and ${\dot{\varepsilon }}_{0}$ is the reference strain rate. T is the current temperature, $T_{tr}$ is the reference temperature, and $T_{m}$ represents the melting temperature of the workpiece. 

The J-C damage model has been exploited to model the separation of chips from the workpiece. The damage criterion factor ‘*D*’ is estimated for each element which compares the total equivalent plastic strain in the element with the failure strain. The fracture is initiated when *D* is equivalent to 1. Factor ‘*D*’ can be obtained using Equation (2):

(2) $$D=\sum \frac{\Delta \varepsilon }{\varepsilon_{f}}$$

where Δε is the equivalent plastic strain for the increment and $\varepsilon_{f}$ represents the equivalent strain at fracture that is computed using Equation (3):

(3) $$\varepsilon_{f}=(D_{1}+D_{2}\exp\left(D_{3}\sigma^{*}\right)\left(1+D_{4}\;\ln\left(\frac{\dot{\varepsilon }}{{\dot{\varepsilon }}_{0}}\right)\right)\left(1-D_{5}{\left(\frac{T-T_{\mathrm{room}}}{T_{\mathrm{melt}}-T_{\mathrm{room}}}\right)}^{m}\right)$$

*D_1_* to *D_5_* symbolizes J-C damage parameters for the workpiece material and are calculated experimentally by using tensile and torsion tests. σ* is the proportion of pressure stress to the von-mises stress. The J-C plastic model and damage parameters are listed in Table 3.

In the second phase, the heat transfer analysis is undertaken using ABAQUS/STANDARD and the user subroutine DFLUX to simulate the moving heat source for extracting the steady-state temperatures on the cutting tool. The general heat conduction formulation can be denoted by Equation (4):

(4) $$\rho \;c\;\frac{\partial T}{\partial t}=\dot{Q}+k\nabla^{2}T$$

where ρ is the density, c is the specific heat, and k is the thermal conductivity of the tool. $\dot{Q}$ characterizes the internal heat generation rate which can be neglected in the current scenario. Taking into account the convective heat transfers for the cutting tool, the formulation can be modified as in Equation (5):

(5) $$\rho \;c\frac{\partial T}{\partial t}=\nabla \left(k\nabla T-\rho \;c{\vec{V}}_{\;}T\right)$$

Considering the moving source rotational speed as ω in the z-direction, the heat transfer equation can be updated as Equation (6):

(6) $$\rho \;c\frac{\partial T}{\partial t}=\nabla \left(k\nabla T-\rho \;c{\vec{V}}_{\;}T\right)+\rho c\omega \;\left(y\frac{\partial T}{\partial x}-x\frac{\partial T}{\partial y}\right)$$

The equation of continuity and the final energy balance equation can be represented by Equations (7) and (8).

(7) $$\frac{\partial \rho }{\partial t}+\nabla \left(\vec{\rho V_{\;}}\right)=0$$

(8) $$\rho \;c\frac{\partial T}{\partial t}=\nabla \left(k\nabla T\right)+\;\rho c\omega \;\left(y\frac{\partial T}{\partial x}-x\frac{\partial T}{\partial y}\right)$$

## 3. Results and Discussion

**Figure 2:**
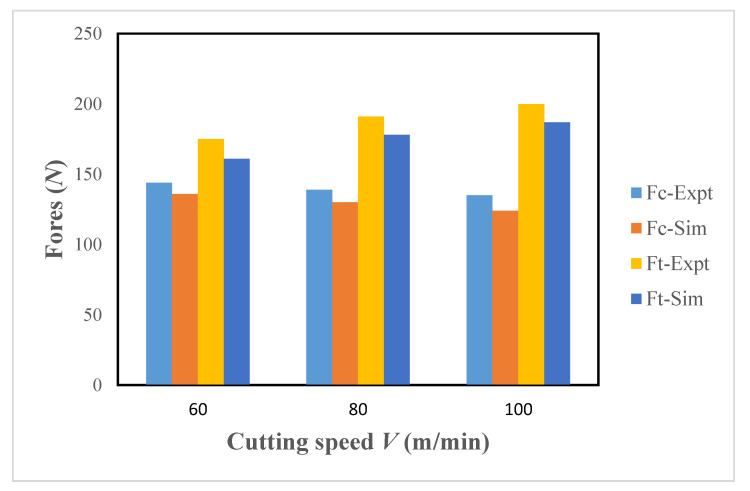
Comparison of the experimental and simulation data for cutting and thrust forces.

Figure 2 provides a comparison of experimental findings and the model simulations. The model has been executed at three cutting speeds, i.e., 60, 80, and 100 m/min using the feed rate of 0.1 mm/rev and the cutting depth equal to 0.2 mm.

The simulation values of the cutting and thrust forces indicate adequate consistency with the experiments and the average error is about 7%. This error can be attributed to various issues associated with model creation. For example, uncertainties related to material flow and damage model parameters in addition to constraints with the chip separation criterion depending on the element deletion method.

**Figure 3:**
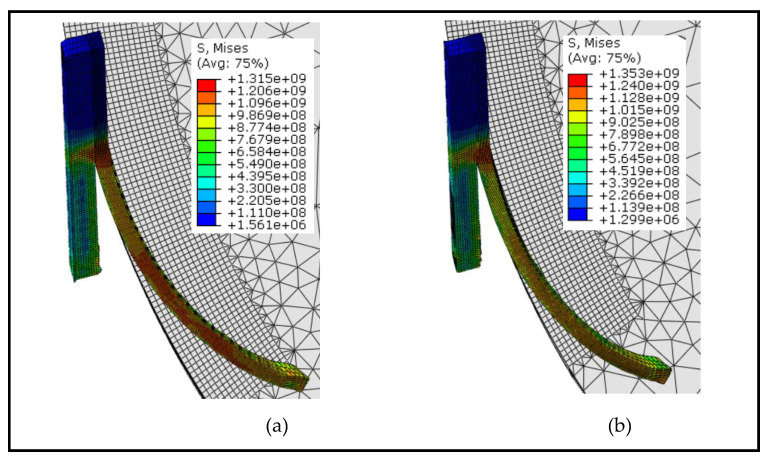
Mises stress (*Pa*) and chip flow at *V* = 100 m/min: (**a**) Rotary tool; (**b**) fixed tool.

**Figure 4:**
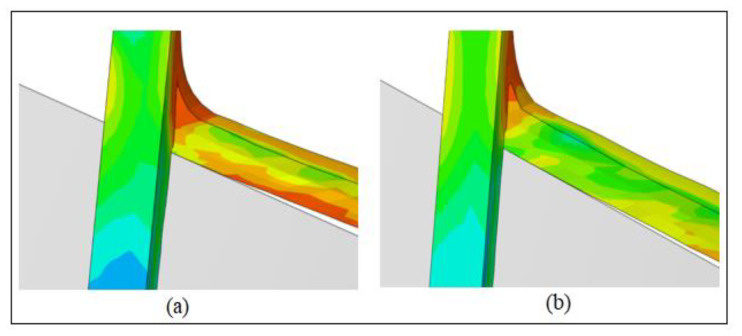
Tool-chip contact length at *V* = 100 m/min: (**a**) Rotating tool; (**b**) fixed tool.

The simulations have also been performed for the fixed tool instance to examine the impact of tool rotation on chip formation and temperature generation. Chip formation and von-mises stress contours based on the rotary and fixed tools at the cutting speed of 100 m/min are displayed in Figure 3. By contrasting the chip flow in both scenarios, it is obvious that, unlike the fixed tool, the chip flow angle with the rotating tool is slightly higher due to the high chip sliding speeds. The magnitude of mises stress in both situations is almost the same. However, the contour pattern is substantially different, and high mises stress regions for the rotating tool case are distributed to the entire chip length owing to a greater chip curl as compared to the fixed tool. Figure 4 includes a comparison of the tool-chip contact length for the rotating and fixed tools. The tool-chip contact length for the rotating tool case is found to be smaller as anticipated, and this implies the significance of SPRT in reducing cutting forces as stated by Dessoly et al. [8]. Figure 4 also demonstrates the variations in mises stress contours as mentioned before.

**Figure 5:**
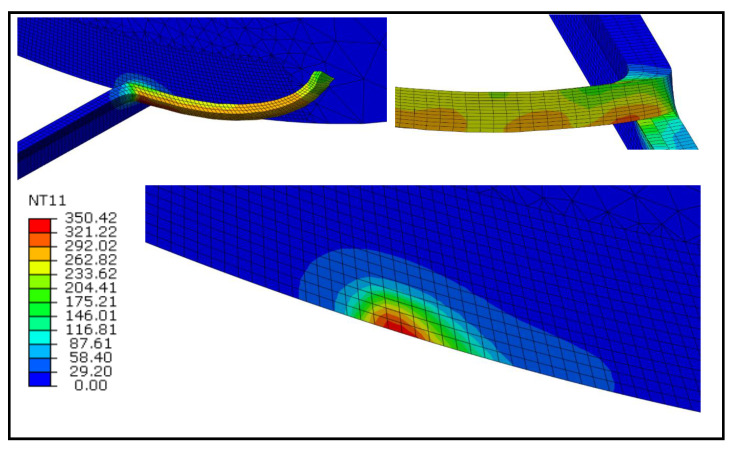
Temperature contours at *V* = 100 m/min for the fixed tool case (°C).

**Figure 6:**
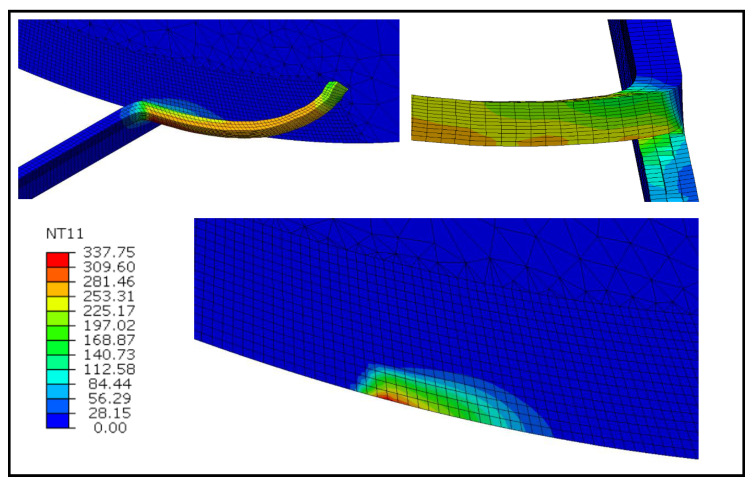
Temperature contours at *V* = 100 m/min for the rotating tool case (°C).

The temperature contours for the fixed and rotating tool cases at the cutting speeds of 100 m/min are illustrated in Figure 5 and Figure 6, respectively. It can be observed that, in both cases, the peak temperature takes place at the tool-chip interface. However, for the fixed tool case, temperatures are around 4% higher. It is apparent by examining the two Figure 5 and Figure 6 that high-temperature regions for the fixed tool case are greater due to the sluggish heat conduction to the tool as opposed to the rotating tool. It is also noticed that in the case of the rotating tool case, the inner surface of the chip is cooler owing to its rapid heat conduction.

**Figure 7:**
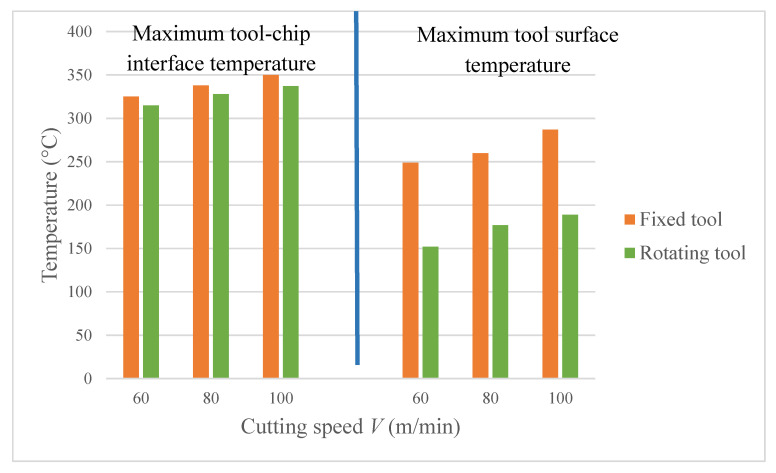
Maximum tool-chip interface and tool surface temperatures at varying cutting speeds for the fixed and rotating tool.

Figure 7 depicts the maximum tool-chip interface and the maximum tool-surface temperatures at varying cutting speeds for the fixed and rotating tool cases. It is evident that similar to the traditional turning the temperature increases with inflation in the cutting speed for all scenarios. The maximum tool-chip interface temperatures for the fixed tool case are slightly higher than for the rotating tool, as can be seen in Figure 7. Nevertheless, there is a substantial difference in the tool surface temperature and an average reduction of about 35% is realized for the rotary tool. It is primarily due to the repeated interchange of the cutting edges identical to the milling cutter, which allows ample time for convection cooling before engagement with the workpiece.

**Figure 8:**
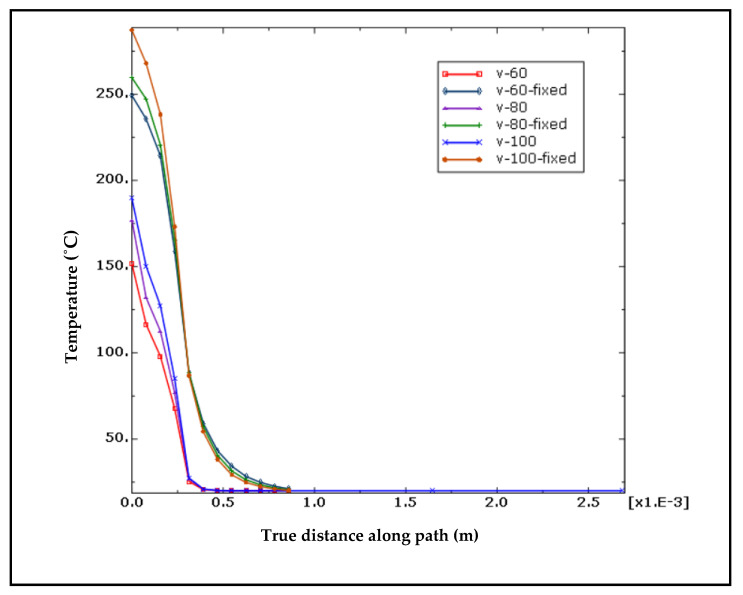
Temperature profiles at the tool cutting edge for different cutting speeds of the fixed and rotating tool.

Figure 8 displays the temperature profiles of the fixed and rotating tool cases for the tool cutting edge at various cutting velocities. From Figure 8, it can be seen that the temperature profiles are quite distinct for both cases. Since higher temperatures for the fixed tool cases are confined to a small region, the temperature curves for different speeds are coincidental at a distance of 0.1 mm from the cutting edge suggesting similar profiles. In comparison, the profiles for the rotating tool cases are rather different and they are coincidental at the distance of 0.25 mm from the cutting edge. This is due to the fact that the high-temperature area is expanding in the direction of rotation of the tool, as revealed in Figure 6. It should also be noticed that the rotary tool displays room temperature at a distance of 0.4 mm from the cutting edge, while the fixed tool shows the same at 0.9 mm from the cutting edge.

**Figure 9:**
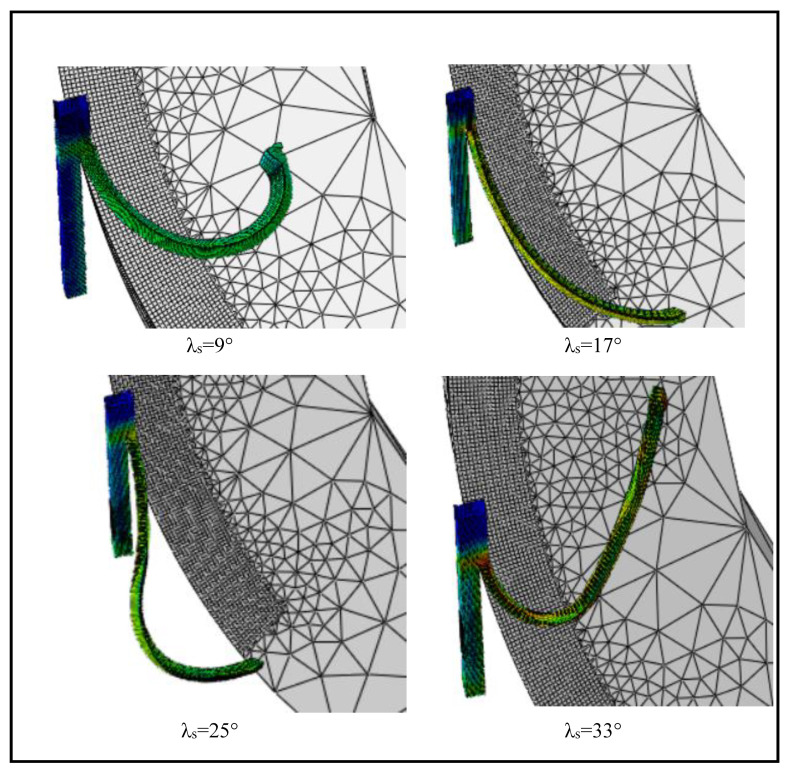
Chip formation at different inclination angles (*V* = 100 m/min).

**Figure 10:**
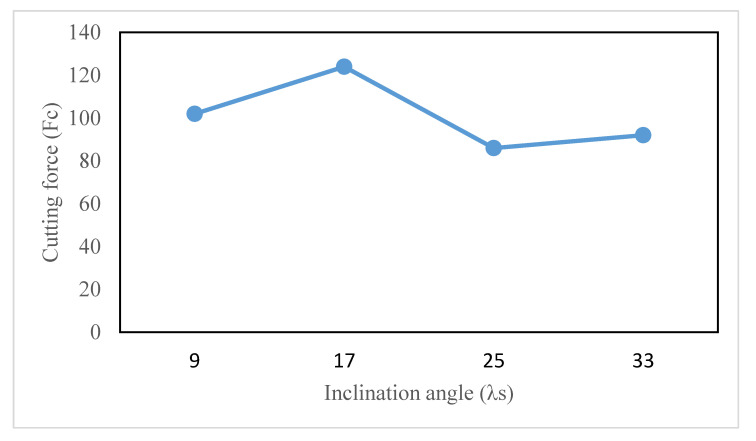
Cutting force variation with different inclination angles (*V* = 100 m/min).

The simulations are also undertaken at the inclination angles of 9°, 25°, and 33°, in addition to the standard inclination angle of 17° to explore the implications of the tool inclination angle (*λ_s_*) on cutting quality. The chip formation at different inclination angles can be viewed in Figure 9. It can be discovered in Figure 9 that the chip flow angle and the tool-chip contact length are strongly influenced by the tool inclination angle. The angle of chip flow initially decreases with the angle of inclination from 9° to 25° and then begins to increase again when the angle of inclination rises further up to 33°. On the other side, the tool-chip contact length is noted to be maximum at 17° and then varies in the fluctuating manner, as presented in Figure 9. As the cutting force is largely dependent on the tool-chip contact length, the pattern is identical to that of the tool-chip contact length, and the highest cutting forces are also found at 17°. Figure 10 depicts the variation in cutting forces with changes in the inclination angle.

**Figure 11:**
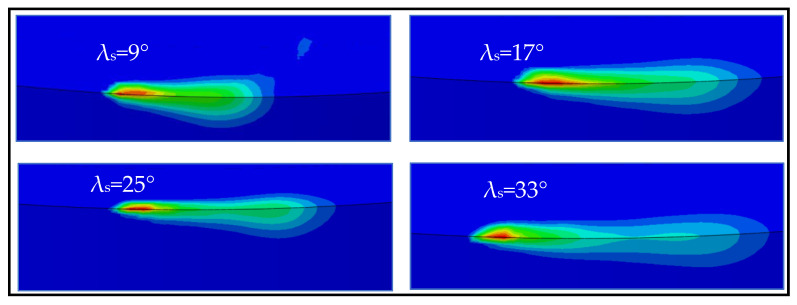
Temperature contours on the tool cutting edge at different inclination angles (*V* = 100 m/min).

**Figure 12:**
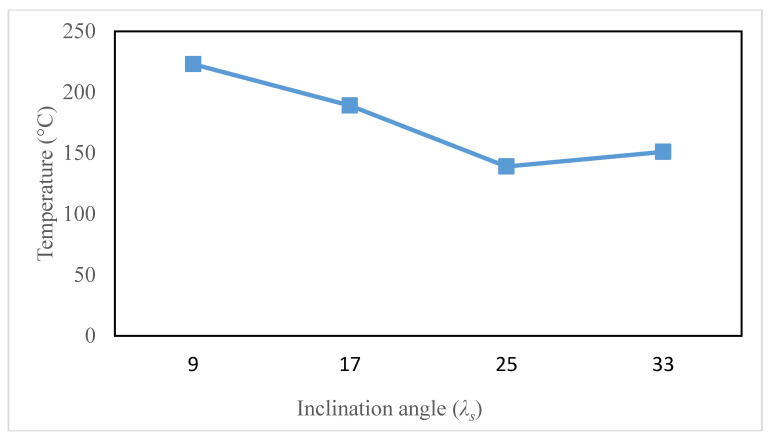
Maximum tool surface temperature at different inclination angles (*V* = 100 m/min).

Figure 11 displays the temperature contours on the tool cutting edge for different inclination angles at the cutting speed of 100 m/min. The temperature contour shape varies considerably as the tool inclination angle is altered from 9° to 33°. This can be shown by the fact that the high-temperature region first grows as the angle of inclination shifts from 9° to 17°, and then tends to decrease as the angle of inclination rises further from 17° to 25°. Due to a large shift in the chip flow angle as presented in Figure 9, the temperature contour shape is markedly different at 33°. However, the maximum tool surface temperature differs similarly and is demonstrated in Figure 12. The maximum temperature first decreases as the angle of inclination increases from 9° to 25°, and then continues to rise as the angle of inclination moves to 33°, i.e., the minimum temperature is located at a 25° inclination. This tendency is consistent with other scholars, such as Shaw et al. [7] and Dessoly et al. [8].

**Figure 13:**
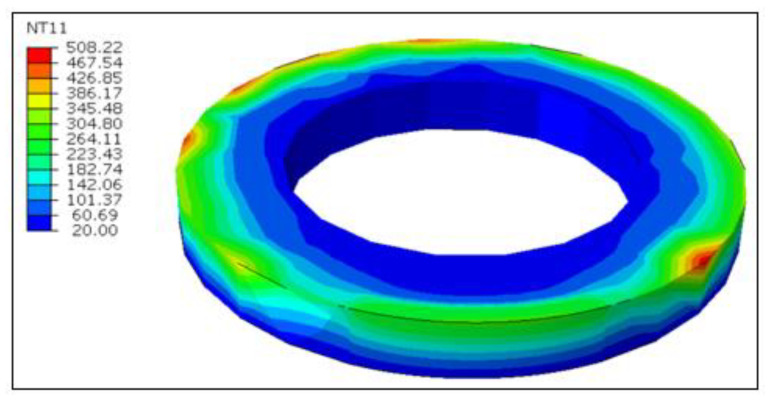
Steady-state tool surface temperature at *V* = 100 m/min for the rotary tool (°C).

**Figure 14:**
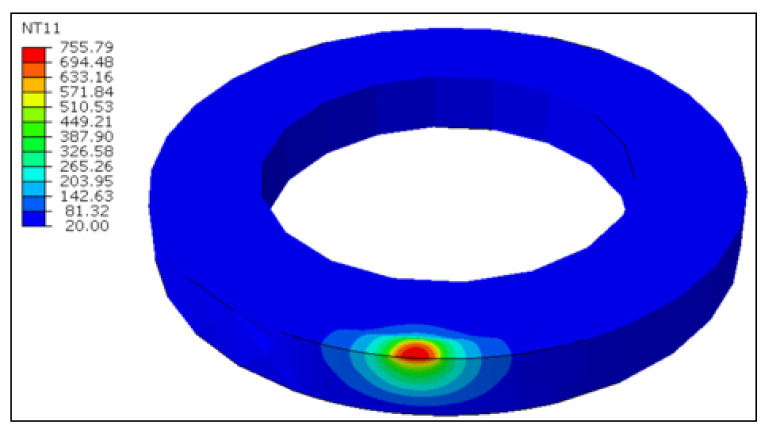
Steady-state tool surface temperature at *V* = 100 m/min for the fixed tool (°C).

The DFLUX subroutine in ABAQUS/STANDARD can be used to retrieve the steady-state tool temperature as mentioned earlier. Figure 13 represents the steady-state temperature of the rotary tool at the cutting speed of 100 m/min and an inclination angle of 17°, following five complete rotations of the heat source. The highest tool temperature on the cutting edge is recorded to be 508 °C, whilst the tool edge average temperature is around 264 °C. The steady-state temperatures for the fixed tool scenario can be interpreted in Figure 14 at the same cutting speed that the elevated temperatures at the tool-workpiece contact area in the fixed tool are limited to a small region, as compared to the rotating tool. In fact, the temperatures are greater due to the persistent interaction of the same cutting edge for continuing contact. Therefore, the rotating tool has displayed noticeable advantages in terms of tool performance and workpiece quality as compared to the fixed tool in hard turning.

## 4. Conclusions

The 3D FE model has been established to examine the hard turning of AISI 51200 hardened steel by utilizing a self-propelled cutting tool. The model is built by incorporating the explicit coupled temperature displacement analysis in the existence of plausible boundary conditions. The model is implemented to estimate the cutting forces, chip flow, stresses, and the temperature distribution on the cutting tool and the part. Since the coupled temperature displacement analysis is computationally expensive, this model cannot be utilized to project the steady-state temperatures on the cutting tool. Therefore, the heat transfer analysis is accomplished using ABAQUS/STANDARD to simulate the steady-state temperatures on the cutting tool and the heat flux. The cutting forces of the model are correlated with experimental data, and a good agreement has been identified. The following inferences can be drawn out from the investigation:

- The 3D FE model established for hard turning using SPRT predicts cutting forces with an acceptable degree of precision.
- The tool-chip contact length for the rotary tool case is observed to be lower due to the variations in the chip flow angles.
- Temperatures are higher for the fixed tool case in the chip forming analysis owing to a slow heat conduction compared to the rotating tool.
- Tool surface temperatures for rotating tools are estimated to be approximately 35% lower than the fixed tools.
- The tool inclination angle has a major effect on various output variables such as chip formation, cutting forces, and temperatures.
- Cutting forces are reported to vary in a fluctuating manner for the range of inclination angles explored. The minimum cutting force is observed at the inclination angle of 25° corresponding to the lowest contact length of the tool-chip.
- Tool surface temperatures decrease initially and then increase with the angle of inclination. The lowest tool temperature is also located at a 25° angle of inclination.

Finally, it can be concluded that the comparison of steady-state temperature profiles for the rotating and fixed tool cases demonstrates the strengths of the rotating tool. The rotating tool case has a low and uniform temperature distribution, which improves the tool performance and surface integrity of the workpiece.

## Figures and Tables

**Table 1:** Workpiece and cutting tool properties.

	Workpiece (AISI 51200)	Cutting Tool (TiN Coated Carbide)
Density (kg/m^3^)	4370	3500
Young’s Modulus (GPa)	408	800
Thermal conductivity (W/m/°C)	30	173
Specific heat (J/Kg/°C)	706	508

**Table 2:** Variables of cutting and tool geometry for the finite element (FE).

Cutting Parameters	
Speed	60, 80, and 100 m/min
Feed rate	0.1 mm/rev
Depth of cut	0.2 mm
**Cutting Tool Geometry**	
Rake angle	−15°
Clearance angle	5°
Inclination angle	17°
Diameter	27 mm
Edge radius	0.05 mm

**Table 3:** Johnson-Cook’s flow and damage parameters.

J-C Flow Model	*A* (MPa)	*B* (MPa)	*C*	*n*	*m*
	688.17	150.82	0.043	0.336	2.77
**J-C Damage Model**	***D_1_***	***D_2_***	***D_3_***	***D_4_***	***D_5_***
	0.05	3.44	−2.12	0.002	0.61
